# Performance of standard procedures in detection of EGFR mutations in daily practice in advanced NSCLC patients selected according to the ESMO guideline: a large Caucasian cohort study

**DOI:** 10.1186/s40247-014-0009-0

**Published:** 2014-09-11

**Authors:** Inge Hantson, Christophe Dooms, Eric Verbeken, Peter Vandenberghe, Liesbet Vliegen, Tania Roskams, Sara Vander Borght, Kris Nackaerts, Isabelle Wauters, Johan Vansteenkiste

**Affiliations:** Respiratory Oncology Unit, Department Pulmonology, University Hospitals KU Leuven, Leuven, Belgium; Department of Clinical and Experimental Medicine, Lab for Pulmonology, University of Leuven, Leuven, Belgium; Pathology, Translation Cell and Tissue Research, University Hospitals KU Leuven, Leuven, Belgium; Human Genetics, Molecular Diagnostics, University Hospitals KU Leuven, Leuven, Belgium

**Keywords:** Predictive factors, Real-time quantitative polymerase chain reaction (rt-qPCR), EGFR mutation, Endobronchial ultrasound, Biopsy methods

## Abstract

**Background:**

ESMO consensus recommends EGFR mutation testing in never/former light smokers (<15 pack-years) or patients with non-squamous NSCLC. The aim of this work was to determine the frequency and clinical predictors of EGFR mutations, and the role of specimen sampling tests, in Caucasian standard practice setting.

**Methods:**

We screened 297 patients according to this consensus. Mutational analysis of EGFR was performed using the Therascreen EGFR RGQ PCR mutation kit. Clinical and pathological correlative data were collected.

**Results:**

An EGFR activating mutation was found in 32 patients (11%), twelve exon 19 deletions, two exon 18 and eighteen exon 21 point mutations. Most were in females, but half were in smokers. Negative TTF-1 staining had a very strong negative predictive value (all except one patient had TTF-1 positive adenocarcinoma). Both biopsies as well as cytology specimens (mainly EBUS-TBNA) did well: 24 mutations in 213 biopsy samples (11.2%) and 8 in 84 cytology samples (9.5%), respectively. The Therascreen acted as a sensitive test in all types of samples: 7 activating mutations were found in samples rated to have <5% of tumour cells, and there were only 4 test failures in the whole series.

**Conclusion:**

In this Caucasian standard practice NSCLC cohort, tested according to the ESMO consensus, activating EGFR mutation occurred in 11% of the patients. Half of these were in former/current smokers. With our sampling technique and use of the Therascreen kit, EBUS-TBNA cell blocks performed as good as biopsies.

**Electronic supplementary material:**

The online version of this article (doi:10.1186/s40247-014-0009-0) contains supplementary material, which is available to authorized users.

## Background

About 85% of all lung cancer patients have non-small cell lung cancer (NSCLC), and the majority presents with advanced stage disease at the time of diagnosis. The standard of care for these patients is platinum-based doublet chemotherapy [1]. This `any platinum doublet fits all’ strategy results in a median overall survival of 8 to 10 months and a 1 year survival rate of about 33%.

Different strategies to prolong survival have been developed. Besides customisation of chemotherapy according to histological subtype [2], and the use of maintenance therapy in order to achieve prolonged tumour control [3], the most important change during the last decade was the introduction of treatment guided by the tumour's genetic profile.

The epidermal growth factor receptor (EGFR) tyrosine kinase inhibitors (TKIs) heralded this evolution, when very pronounced and durable responses to these agents were noted early in this century [4]. A few years later, somatic mutations in the EGFR gene were discovered in these highly responsive tumours. They affect the tyrosine kinase domain of the EGFR - involving exons 18 until 21 - and lead to constitutive activation of the receptor, independent of ligand binding. The tumour becomes highly dependent on this pathway, and thus very sensitive to blockade of this pathway by TKIs [5]. Recent randomised trials demonstrated that the presence of an EGFR activating mutation was the best predictive factor for response and progression-free survival (PFS) to EGFR TKIs when compared to platinum doublet chemotherapy in the first-line therapy of advanced NSCLC [6],[7]. Given this strong benefit of EGFR-TKIs in patients with a tumour with an EGFR activating mutation (EGFR mut + tumour), molecular profiling became necessary in the assessment of stage IV NSCLC.

EGFR mutations are known to be associated with clinical characteristics such as never-smoking status, female gender, adenocarcinoma histology and South-East Asian ethnicity [8]. Adenocarcinoma is by far the most common NSCLC histology in South-East Asia, and about 40% of these tumours are EGFR mutant [9]. The EGFR mutation occurrence is much lower in Caucasian populations. As both gefitinib and erlotinib are now registered in Europe for the treatment of patients with an EGFR mut + tumour, selection criteria to identify which patients are most likely to have an EGFR mutation are needed. Based on the South-Asian experience and the occurrence of the different NSCLC histologies in Europe, a European guideline (ESMO consensus [1]) recommends EGFR mutation testing in never-/former light (<15 pack-years) smokers or patients with non-squamous NSCLC.

As a consequence and in contrast with 10 years ago, precise histological subtyping and EGFR testing is now mandatory. Many colleagues of the multidisciplinary team involved in the diagnostic flow of NSCLC - pulmonologists, surgeons and radiologists - need to optimise biopsy samples (size, content of tumour cells).

Until now, the most common diagnostic procedure for the diagnosis of NSCLC was bronchoscopy, which may however not always provide enough tissue for molecular analysis. We therefore evaluated the performance of different small tissue samples obtained during bronchoscopic procedures, such as bronchial biopsies and endobronchial ultrasound guided transbronchial needle aspirations (EBUS-TBNA), in the molecular diagnostic setting of NSCLC. Study aims were (1) the frequency of EGFR mutations (exon 18-21) in a large Caucasian cohort; (2) clinical and pathological predictors; (3) the sensitivity of the Therascreen kit; and (4) the performance of cytology versus biopsy samples for mutation analyses.

## Methods

### Study subjects

All consecutive EGFR mutation analysis reports in the period from September 2010 until December 2011 included were retrieved from the molecular genetics database. During this period, our local policy was to order EGFR mutation testing in all patients diagnosed with advanced NSCLC, either of adenocarcinomas or not-otherwise specified (NOS) histology, irrespective of smoking history, or in those rare patients with squamous cell carcinoma with a negative or light (<15 pack years) smoking history.

### Methods

The clinical data of the patients were retrieved retrospectively from the clinical records. We categorised male and female patients in age, grouped as less than 60 years old, between 60 and 70 years and older than 70 years. Smoking history of the patients was obtained at baseline, and patients were categorised as never-smokers, former smokers (stop >1 year), or current smokers (stop < 1 year or still smoking). We also looked at the number of pack years.

Each patient had one sample in this analysis, either a cytological or histological one. Most of the histology samples were bronchial biopsies, with a few resection specimens, and most of the cytology samples were EBUS-TBNA samples. In each patient, at least 4-5 bronchial biopsies using a 2 mm forceps (Boston) were taken or at least 3 representative needle aspirations using a 22Ga EBUS-TBNA needle (Olympus) until a sufficient amount of microcores was obtained as judged by the operator. Samples underwent fixation in 6% neutral-buffered formalin, during 6-12 hours for bronchial biopsies and 8-18 hours for surgical specimens. EBUS-TBNA samples were collected in Cytorich Red fluid, formalin fixed for 6-12 hours and paraffin-embedded in a cell block.

Immunohistochemistry (IHC) for EGFR was carried out using a mixture of two mouse monoclonal antibodies, clone NCL-L-EGFR-384 (1:100, Novocastra) and clone E30 (1:50, Dako) in Dako's EnVison FLEX Antibody Diluent (Dako) on a Dako Autostainer Plus (Dako Denmark) according to the manufacturer's recommendations. EGFR expression was reported as positive in case of membranous brown staining in at least 10% of the cells. TTF-1 was also immunostained using a mouse monoclonal antibody (clone 8G7G3/1, ready-to-use, Dako), following the same procedure as described for EGFR. Tumours were considered TTF-1 positive in case of nuclear staining of neoplastic cells.

For EGFR mutation analysis, one H&E slide followed by 10 serial unstained sections (4-μM thick) and a final H&E were prepared from the paraffin block and both H&E sections were evaluated for the presence and amount of tumour cells by an experienced pathologist. After macro-dissection and deparaffinisation, the tissue was digested overnight with proteinase K. DNA extraction was done using the Maxwell16 FFPE tissue LEV DNA purification kit on the Maxwell16 instrument (Promega Corporation, Madison, WI) according to manufacturer's instructions. Mutational analysis of EGFR was performed using the Therascreen EGFR RGQ PCR kit (produced by Qiagen) on the LightCycler 480II instrument (Roche). The Therascreen assay is a sequence of two techniques, ARMS and Scorpion, to detect mutations in real-time PCRs with a reported detection limit of 1% mutant alleles in DNA from tumour tissue. This kit allows the detection of the nineteen deletions between 2235 and 2258 in exon 19, three insertions in exon 20 and the point mutations G719A/S/C (exon 18), S768I (exon 20), T790M (exon 20), L858R (exon 21) and L861Q (exon 21). The test has both EU as well as FDA approval.

### Ethics

This research was approved as a retrospective, non-interventional, study by the local Institutional Review Board of the University Hospitals KU Leuven.

## Results and discussion

### Clinical characteristics

From September 2010 until December 2011, a total of 297 NSCLC samples were analysed (Table 1). The population consisted of 113 females (38%) and 184 males (62%). The median age was 68 years (SD 11), eighty patients were younger than 60 years, 99 were between 60 and 70 years old and 118 were older than 70 years. All patients were Caucasians. Smoking status was known in 248 patients, 40 (16%) were never smokers, 109 (44%) were former smokers (stop >1 year) and 99 (40%) were current smokers.

**Table 1:** **Clinical and pathological patient characteristics**

	Patients (N)	EGFR mut + (N)	EGFR mut + (%)
Total group	297	32	11
Gender			
Female	113	26	23
Male	184	6	3
Age			
<60	80	7	9
60-70	99	10	10
>70	118	15	13
Smoking			
Never	40	16	40
Former	109	8	7
Current	99	7	7
Missing	49	1	-
Specimen			
Cytology	84	8	10
Biopsy	213	24	11
Tumour histology			
Adeno	274	31	11
NSCLC-NOS	9	1	11
Squamous	14	0	0
EGFR IHC			
Positive	254	29	12
Negative	9	0	0
Missing	34	3	-
TTF-1 IHC			
Positive	171	29	17
Negative	45	1	2
Missing	81	2	-

EGFR mut+: presence of activating EGFR mutation; N: number; NSCLC-NOS: non-small cell lung cancer not otherwise specified; IHC: immunohistochemistry; TTF-1: thyroid transcription factor 1.

### Pathology

We examined 84 (28%) cytology samples and 213 (72%) biopsy samples. Out of the 84 cytology specimens, 80 were EBUS-TBNA cellblocks, and 4 were pleural cytology. Biopsy samples were 114 bronchial biopsies, 48 archived resection specimens, 12 lymph nodes (7 mediastinoscopy, 5 supraclavicular), 17 pleural biopsies, 11 trans-thoracic lung tumour punctures, and 11 metastatic sites (2 bone, 2 brain, 2 liver, 4 soft tissue, 1 adrenal gland).

Two hundred and seventy-four patients had adenocarcinoma (92%), fourteen had squamous cell carcinoma (5%) and nine had NSCLC-NOS (3%). EGFR immunohistochemistry was known for 263 patients, and was negative (i.e. <10% membranous staining) in only 9 patients. TTF-1 status was known in 216 patients, and was positive in 171 patients (79%).

**Figure 1:**
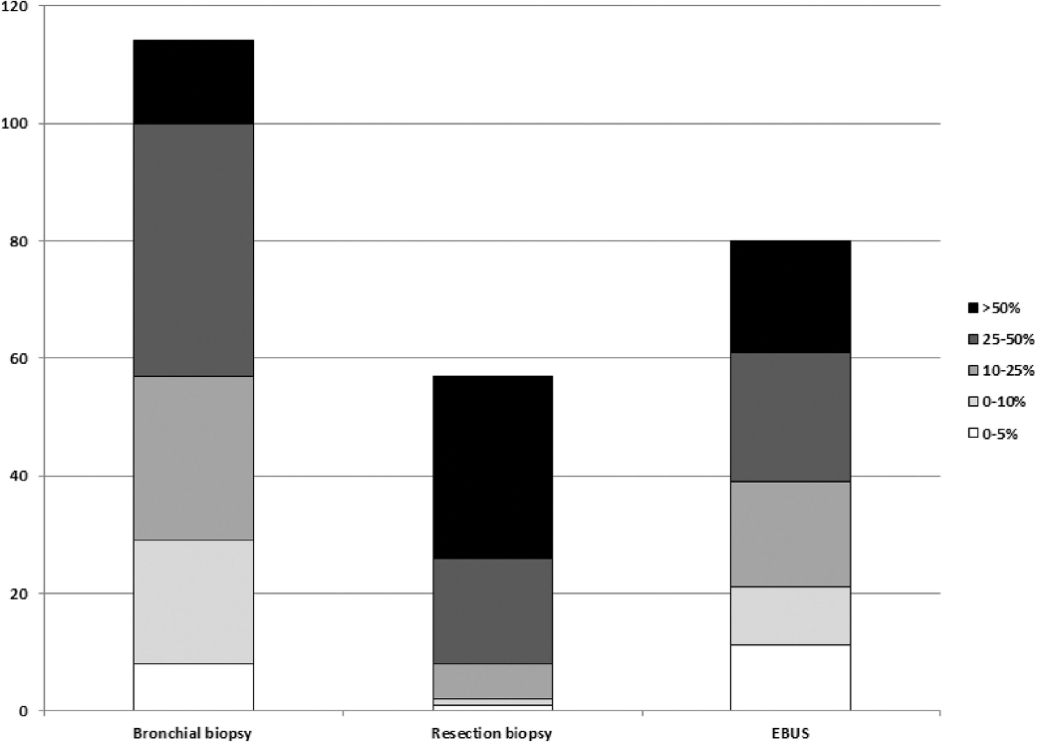
**Cumulative numbers of tumour cell content in different samples.** EBUS: needle aspiration in lymph node guided by endobronchial ultrasound guided procedure.

The percentage of tumour cells was described in 293 samples. Fifty-seven samples had less than 10% tumour cells (19%), 152 samples had between 10 and 50% tumour cells (52%) and 84 samples had more than 50% tumour cells (29%). There was no significant difference in percentage of tumour cells between the cytology specimens and the biopsy samples. Out of 84 cytology specimens, 22 had less than 10% tumour cells (26%) versus 35 samples in 213 biopsy specimen (16%). Out of 84 cytology specimens, 41 had between 10 and 50% tumour cells (49%) versus 111 samples in 213 biopsy specimens (52%). Out of 84 cytology specimens, 21 one had more than 50% tumour cells (25%) versus 63 samples in 213 biopsy specimens (30%) (Figure 1).

### EGFR mutation test results

Activating EGFR mutations (EGFR mut+) were found in 32 patients (11%, Table 1): twelve patients had exon 19 deletions (38%), two patients had G719A/S/C point mutations in exon 18 (6%) and eighteen patients had exon 21 point mutations (56%), of which fifteen L858R mutations and three L861Q mutations. Inhibitory exon 20 insertion mutations were detected in five patients (2%).

Most mutations were found within the elderly population. Thirteen percent mutations in patients older than 70 years, 10% mutations in patients age between 60 and 70 years and 9% activating mutations in patients younger than 60 years.

EGFR mutation positive patients were mostly female (81%), 26 females versus 6 males. All but one were adenocarcinomas (97%), no activating mutations were found in squamous cell carcinomas. EGFR-mut + were clearly more present in never smokers (16/39, 41%) than in current (7/97, 7%) or former smokers (8/108, 7%).

All but one EGFR-mut + patients had immunohistochemistry (IHC) >50% for EGFR. This patient had 25% IHC + for EGFR. We found no mutations in samples with low (≤10%) EGFR-IHC. All but one EGFR-mut + patients had TTF-1 positivity.

We found 8 EGFR-mut + in 84 cytology specimens (10%) and 24 EGFR-mut + in 213 biopsy samples (11%).

Despite the fact that 21 out of 289 samples (7%) had low tumour cell percentage (0-5%), we found 7 activating mutations in these (33%). The Therascreen kit failed 4 times (1%). One failure was a bone biopsy, two had low (5%) tumour percentage and one was a bronchial biopsy with 25% of tumour cells. This confirms that the Therascreen kit is a very sensitive test, with low test failures, mostly when the tumour cell count was rated <5% by the pathologist.

### Discussion

In the time interval September 2010 - December 2011, we selected advanced NSCLC patients for EGFR mutation screening in exon 18-21 by a PCR based test according to the ESMO criteria [1]. In our 297 patients, the testing was successful in all but four, and we detected an activating mutation in 11%. Not surprisingly, activating mutations were more frequently reported in women, never smokers, and pure adenocarcinoma.

Based on this series and review of other European findings, we believe that 9 to 12% may well be the real frequency of activating EGFR mutations in advanced NSCLC in Europe (Tables 2 and 3). Our frequency finding is in line with earlier Spanish work (12% [10], and very recent data from routine practice in e.g. the UK (9.7% [11], 10% [12]), Lithuania (9.7% [13]), and the Netherlands (8.4% [14]).

**Table 2:** **Demographics in European series on EGFR mutations testing**

Series	Year	Origin	N	Stages	Further description
Cortes-Funes et al. [10]	2005	Spain	83	Advanced	Failing 1st line/TKI therapy
Marchetti et al. [15]	2005	Italy	860	Stage I-II-III	Resection specimen
Rosell et al. [19]	2009	Spain	2105	Advanced	-
Penzel et al. [21]	2011	Germany	1047	All	-
Helland et al. [16]	2011	Norway	240	Stage I-II-III	Resection specimen
Vincenten et al. [20]	2012	Netherlands	810	All	-
Smits et al. [14]	2012	Netherlands	778	Advanced	-
Leary et al. [12]	2012	England	144	All	-
Pennycuick et al. [11]	2012	England	215	All	Non-squamous NSCLC
Vaguliene et al. [13]	2012	Lithuania	103	Advanced	Non-squamous NSCLC
Cadranel et al. [22]	2012	France	522	All	Failing 1st line/TKI therapy
This series	2012	Belgium	297	Advanced	ESMO guideline selected

N: number; TKI: tyrosine kinase inhibitor; NSCLC: non-small cell lung cancer; ESMO: European Society of Medical Oncology.

**Table 3:** **Results in European series on EGFR mutation testing**

Series	Adeno (%)	Female (%)	Never-smokers (%)	EGFR mut + (%)	SQCC (N)
Cortes-Funes et al. [10]	51	30	24	12	0
Marchetti et al. [15]	44	13	13	4.5	0
Rosell et al. [19]	85	39	29	16.6	0
Penzel et al. [21]	NA	NA	NA	14.7	0
Helland et al. [16]	59	48	6	6.6	2
Vincenten et al. [20]	65	49	NA	14	0
Smits et al. [14]	80	46	10	8.4	0
Leary et al. [12]	67	59	NA	10	0
Pennycuick et al. [11]	NA	57	NA	9.7	0
Vaguliene et al. [13]	63	25	21	9.7	0
Cadranel et al. [22]	65	32	18	14	0
This series	92	38	16	11	0

Some series have reported remarkably lower frequencies. These were investigations on resection specimens, an earlier one from Italy with a 4.5% occurrence [15], and a recent one from Norway (6.6% [16]). In another of our series on resection specimens, the frequency was lower as well at 7.0% (personal communication C. Dooms). This could be due to truly lower occurrence in early resectable stages, although this seems less likely. It has been postulated that mutations in the tyrosine kinase domain of EGFR are early events in lung carcinogenesis, already present in 50% of precancerous conditions such as atypical adenomatous hyperplasia, as well as in normal lung tissue surrounding a tumour [17]. More probable, several technical factors may play a role, as resection specimens usually don't suffer from *tissue quantity* but may suffer from *tumour DNA quality*. Indeed, the thoroughness of formalin fixation (which may be less good in large resection specimens) and the duration of storage (with DNA degradation over time) may affect the quality of extracted DNA and render the sample less suitable for mutation analysis. Williams et al. illustrated that as much as one artificial mutation per 500 bases may be recorded in the analysis of formalin-fixed material, because of formalin damaging or cross-linking cytosine nucleotides [18].

Several European studies also found higher frequencies than our study, but (unconscious) selection for testing based on clinical factors may have played a role in these findings. As a reminder and for comparison, in the hitherto largest - predominantly European - study in advanced NSCLC [2], there were 30% female patients and 14% never-smokers. In the more recent Spanish findings in the context of a randomised controlled trial, occurrence of exon 19 or 21 mutations was 16.6% [19]. In this series, there were 39% females, 29% never-smokers, and 75% never- and former smokers combined. In a recent Dutch series [20], the mutation rate was 14%, again with 49% females. In the very large German series, no information on gender or smoking is provided, but as the screening program was set up for the introduction of 1^st^ line gefitinib in the treatment of advanced NSCLC, some pre-selection on clinical factors may again have been in place [21]. The series from France had a very different aim than the studies above [22], in particular examining the feasibility of EGFR and KRAS testing in the quite different context of use of erlotinib for 2^nd^ or 3^rd^ line treatment for all NSCLC histologies. In this group consisting of 86% never- or former smokers, the EGFR mutation rate was 14%.

We found no EGFR mutations in our never- or light-smokers with squamous cell carcinoma. This is consistent with previously published data. All European series found no mutations in squamous cell carcinoma, except for the Norwegian study, where two activating mutations were reported in surgical resection specimens. In a recent interesting pathology review study, all specimens with diagnosis of EGFR/KRAS-mutant squamous cell cancer were reassessed with modern techniques to differentiate squamous from adenocarcinoma differentiation [23]. Detailed morphologic and immunohistochemical study resulted in reclassification of 10 cases as adenosquamous cancer and 5 as poorly differentiated adenocarcinoma morphologically mimicking squamous cell cancer.

To optimize genetic testing in NSCLC, it is important to maximize the amount of tumour cells within the tissue sample. We investigated the tumour cell percentage on cytology and biopsy samples, and its effect on PCR-based detection of EGFR mutations. There was no significant difference between our bronchial biopsy samples and cell blocks of the cytology samples obtained by EBUS-TBNA. Our findings are in line with another recent multicentre series that examined if EBUS-TBNA cytology specimens are suitable for phenotyping and genotyping of NSCLC. A brief report from the Netherlands in 2010 suggested that specimens from FNA of the left adrenal gland were suitable for EGFR mutation analysis in 77% of the cases [24]. In a comparative study of biopsy and fine needle aspiration samples, the latter yielded lower DNA amounts but a comparable success rate in EGFR mutation analysis [25]. In a recent large series, EGFR mutation analysis was possible on cell block samples of 107/119 (90%) patients in whom it was requested [26].

ESMO guidelines advocate an EGFR TKI as the preferred first-line treatment in NSCLC patients whose tumour harbours an activating EGFR mutation [1]. A remaining practical controversy is how waiting time for EGFR mutation analysis should be balanced with delay of start of treatment with platinum doublet chemotherapy, especially as the overall survival with both strategies has hitherto not been shown to be significantly different [27]. Clinical features are related to but not sufficiently reliable to estimate the presence of EGFR mutations. Consequently, a rapid test with high negative predictive value (NPV) for EGFR mutations is welcome. TTF-1 IHC seems to fill this gap. Indeed, in series where this relationship is reported (Table 4), the NPV varies between 96 and 100%, although the NPV may be overestimated, as all series have a rather low prevalence of EGFR mutation. As many advanced NSCLC samples nowadays undergo IHC staining for the differentiation between adenocarcinoma or squamous cell carcinoma [28], TTF-1 staining is included or a little extra with great potential for the clinician: if the TTF-1 IHC is negative, doublet chemotherapy can be started. If an EGFR mutation is eventually detected, the EGFR-TKI can be given thereafter. Moreover, there is a good biological rationale for this high NPV, as TTF-1 expression is found in the terminal respiratory unit (TRU) cells, where peripheral tumours with a non-mucinous lepidic or papillary predominant pattern arise. Both types are known to harbour more frequently activating EGFR mutations [29].

**Table 4:** **Correlation between TTF-1 immunohistochemistry and presence of EGFR activating mutation**

Series	EGFR mut + (N)	EGFR mut- (N)	
Vincenten et al. [20]			NPV
TTF-1 +	105	403	96%
TTF-1 -	9	228	
Leary et al. [12]			NPV
TTF-1 +	15	40	100%
TTF-1 -	0	15	
This series			NPV
TTF-1 +	29	140	98%
TTF-1 -	1	43	
Pooled results			NPV
TTF- 1 +	149	583	97%
TTF-1 -	10	286	

EGFR mut+: presence of activating EGFR mutation; N: number; TTF-1: thyroid transcription factor 1; NPV: negative predictive value.

## Conclusion

Both biopsies and cell blocks of EBUS-TBNA are suitable samples for EGFR mutation analysis when a sensitive method such as the Therascreen EGFR RGQ PCR mutation kit is used. We document an 11% frequency of EGFR activating mutations in our Caucasian standard practice NSCLC cohort, selected according to the ESMO consensus guideline.

## Authors’ original submitted files for images

Below are the links to the authors’ original submitted files for images. Authors’ original file for figure 1
