# Supplementary figures and images for: Performance of standard procedures in detection of EGFR mutations in daily practice in advanced NSCLC patients selected according to the ESMO guideline: a large Caucasian cohort study
Source: Transl Respir Med. 2014 Sep 11;2:9. doi: 10.1186/s40247-014-0009-0 (PMC4173071; doi:10.1186/s40247-014-0009-0)

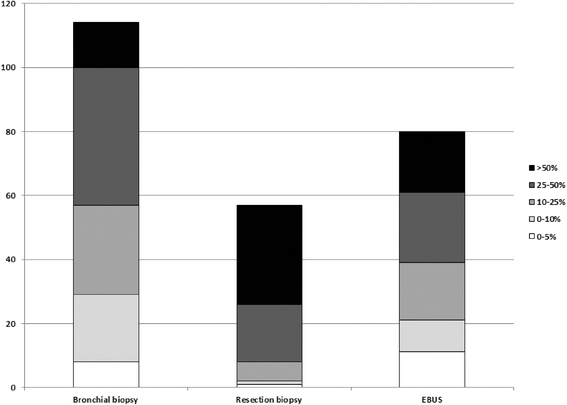

Supplement: Supplementary file 1 — Authors’ original file for figure 1 [file 40247_2014_9_MOESM1_ESM.gif]
